# On a Form of Indigestion and Its Treatment

**Published:** 1893-07-22

**Authors:** A. Hanbury Frere


### ON A FORM OF INDIGESTION AND ITS TREATMENT.

By A. Hanbury Frere, M.B.

The subject of indigestion has occupied the minds of medical men from the earliest times, and our present knowledge of the process of nutrition is the outcome of much laborious work on the part of many of the ablest men throughout the world.

I desire to draw attention to a form of indigestion which is best called duodenal dyspepsia, as it is met with in actual practice.

As far as I have been able to discover, but little has been written on this subject, but I am convinced that its importance and the frequency of its occurrence are far greater than is generally supposed.

Paris* devotes a whole chapter to "Imperfect Digestion in the Duodenum." In Fagge's book, † under "Hepatic Dyspepsia," a condition which is closely allied to "lithaemia," is described, and is in many respects similar to the one under consideration.

Herschell, ‡ in his admirable little work, makes but short reference to duodenal dyspepsia, showing, in my opinion, how much there is yet to be learned in regard to what is perhaps the most important phase in the process of nutrition, and almost the final act of digestion.

The nearer we come to the final act of digestion, the more intricate and subtle are the transformations the food has to undergo. The importance of chymification in the stomach is, in my judgment, nothing to that of chylification in the duodenum, and yet how little attention has been paid to the clinical aspect of cases in which this most delicate and important phase of nutrition is imperfectly carried out.

Such cases are, I believe, generally treated for "stomach" when that viscus is working very well; or for "liver" when that organ is not mainly at fault; or for dyspepsia in its widest sense, without any search after the true source of the trouble. I fear that most digestive derangements are but too often treated in a routine rather than a rational method, though not so much as formerly, and one must rejoice to see that there is a growing tendency to regard the time honoured "pot. bic." as at least only a palliative, and in some cases actually harmful.

There can be little doubt that the duodenum has some important function in nutrition, apart from that of a mere meeting place of the chyme, the bile, and the pancreatic secretion.

Be this as it may, there are certain well defined indications which point to derangement of the duodenum. The patient will complain of pain, at times so severe as to "double him up"; this may be followed by vomiting. We find that this pain comes on three to five hours after a meal, and is situated towards the right side and not at the pit of the stomach. It is generally localised in some portion of the duodenum, and there may be a fulness in this region. The tongue is, as a rule, normal, giving no indication of any gastric derangement, and if it is furred at all, it is only at the back, a condition that one has been taught to associate with derangement of the liver, though I am convinced this is not always so. I believe the tongue to be a most valuable indication in disease to those who know how to interpret its meaning, and in spite of what Dickinson has said to the contrary, I can only say that I have found its indications most valuable, and as is proved by the success of treatment based upon those indications, I have never been deceived in the valuable aid to be obtained by observing the state of the tongue. The bowels are, as a rule, regular, with a tendency to looseness; or there may be alternating diarrhœa and constipation.

From the cases that have come under my notice I cannot attribute duodenal dyspepsia entirely to hepatic derangement, for my patients have, without exception, been more or less active in habit, and not of that sedentary type so often the subjects of "liver."

As to treatment, I am careful to lay down certain rules as to diet, the time and number of meals, and principles to guide in the matter of sleep, rest, and exercise, which cannot be entered into here. Such cases do well on strictly meat diet, until such time as a careful admixture of vegetable food can be taken.

As for drugs, I have found nothing so good as vini colchici in ♏xx. doses, with pot. sulph. (grs.xxx) and a bitter vegetable infusion, and occasionally nux vomica and aloes or cascara sagrada.

I trust I have said enough to draw attention to this not uncommon and very important condition, and one that is often overlooked, but which at the same time is very amenable to treatment.

* "On Diet" (1830). † "Principles and Practice of Med." (2nd edition). ‡ "On Indigestion" (1892).

